# Training and match load in professional rugby union: Do contextual factors influence the training week?

**DOI:** 10.17159/2078-516X/2021/v33i1a9509

**Published:** 2021-05-25

**Authors:** SW West, S Williams, P Tierney, T Batchelor, MJ Cross, SPT Kemp, KA Stokes

**Affiliations:** 1 Department for Health, University of Bath, Bath, UK; 2 Sport Injury Prevention Research Centre, Faculty of Kinesiology, University of Calgary, Calgary, Canada; 3 The Football Association, Burton-on-Trent, UK; 4 Harlequins Rugby Union, Twickenham, UK; 5 Premier Rugby Limited, Twickenham, UK; 6 Rugby Football Union, Twickenham, UK; 7 London School of Hygiene and Tropical Medicine, UK

**Keywords:** athlete management, training load, athlete, injury prevention, performance

## Abstract

**Background:**

Rugby union demands a multifaceted approach to training, given the multiple physical and technical attributes required to play the sport.

**Objectives:**

The aim of this study is to describe the distribution of training throughout the week and investigate how this may be influenced by match-related factors.

**Methods:**

Training load data (session Rating of Perceived Exertion [sRPE], total distance and high-speed running [HSR]) were collected from six professional English rugby teams during the 2017/18 season. Five contextual factors were also recorded including: standard of opposition, competition type, result of previous fixture, surface type, and match venue.

**Results:**

The day prior to matches demonstrated the lowest training load (101 AU (95% CIs: 0–216 AU), 1 047 m (95% CIs:1 128–1 686 m) and 59 m (95% CIs: 0–343 m), respectively), while four days prior to the match demonstrated the highest training load (464 AU (95% CIs: 350–578), 2 983 m (95% CIs: 2 704–3 262m) and 234m (95% CIs: 0–477m), respectively). Of the five contextual factors, competition type was the only variable that demonstrated greater than trivial findings, with training before European fixtures the lowest stimulus across the four different competition types. Standard of opposition, previous result, surface type and venue had only trivial effects on training load (effect sizes = −0.13 to 0.15).

**Conclusion:**

Future studies should outline the distribution of other training metrics, including contact and collision training. This study provides a multi-club evaluation that demonstrates the variety of loading strategies prior to competitive match play and highlights competition type as the most influential contextual factor impacting the average training load.

Training for sport is the process of applying stressors to the body to improve physical and tactical capacity and sporting performance.^[1, 2]^ Training loads are prescribed with the aim of inducing physiological change and must be applied periodically to align with the individual needs of the athlete, the tactical requirements, and the competitive demands of the sport. When preparing for the demands of rugby union, a multifaceted programme must be used, given the broad range of physical and technical attributes required.^[3]^ Such a programme typically includes components of rugby skills (full, semi- and non-contact), as well as conditioning strategies.^[4]^

Athlete load monitoring has become a regular part of most professional sporting environments, with measures of both external and internal load typically used to determine the ‘dose-response’ relationship to training and matches.^[4, 5]^ In rugby union, a broad range of measures are used, including session Rating of Perceived Exertion (sRPE) and Global Positioning Systems (GPS).^[6]^ On average, Premiership rugby players in England undertake 6 hrs 48 min (95% CIs: 6 hrs 30 mins − 7 hrs 4 mins) of training each week ^[7]^; however, how this volume is distributed across the training week, and the types of training undertaken are not well described. In football, the distribution of load across the week demonstrates that matches represent the highest ‘dose’, with the load declining in the three days prior to each fixture.^[8]^ The periodisation of weekly training in rugby union is likely to be similar; however, studies of this type in rugby union are scarce. Further to this, studies in other sports outlining the weekly training structure often include just one team and therefore represent the preparation strategies of just that one team^[8, 9]^, meaning generalisability to the wider playing population is low. The lack of published data in this area is likely due to the reluctance of elite teams to publish data to retain a competitive advantage.^[8]^

While training load is individually prescribed and specific to the timing of the match, a number of contextual factors may also play a role in the prescription of training throughout a week. These contextual factors include the standard of the opposition, the result in the previous fixture, match location and competition type.^[10]^ In rugby union, one further contextual factor that has been reported is that of surface type ^[11]^, with several professional teams reporting an alteration to match preparation based on playing on artificial surfaces compared with natural grass. Given the potential influence of these factors on training, this study aims (i) to describe the weekly structure of training using both internal and external measures of training load, and (ii) to investigate the association between contextual factors and weekly training load in a cohort of English professional rugby union clubs.

## Methods

### Participants

During the 2017/18 season, 397 players consented to partake in this study from six English Premiership teams (the highest level of rugby union in England). The participants were provided with information regarding the study before signing individual consent forms as approved by the Research Ethics Approval Committee for Health at the University of Bath (Ref: 15/16 252).

### Procedures

For each of the 397 players in the study, daily training load data were recorded by Sport Science/ conditioning staff at each club. These data were collected as per normal daily monitoring practices. Following an initial pilot study to select common variables^[6]^, two measures were selected to be included as external load markers (high-speed running (HSR) and total distance), as well as one internal load measure, session Rating of Perceived Exertion (sRPE)^[12]^. The clubs included in the study were taken from a wider population of twelve teams across the competition and were selected because they used HSR and total distance derived from the same GPS provider (Catapult, Catapult Sports, Australia). Of the teams included in the study, two did not consistently collect sRPE data on match days and one did not consistently collect total distance data. This meant they were excluded from the analysis for each of those respective variables as the inconsistent collection of data rendered the data unsuitable for inclusion in this study. Only clubs using relative HSR (thresholds defined by a percentage of each individual player’s maximum velocity) were included in the final analysis (due to difficulty comparing relative and absolute values); however, given the desire to not impose time-consuming data collection procedures on the club staff, each club’s own definition of high-speed running was used. This meant a range of thresholds for high-speed running were used, (>60% Vmax for two clubs, >70% Vmax for one club, and 40–70% of Vmax for the final club). The use of differing HSR thresholds across teams did not impact on the main research objectives, as the statistical analyses assessed the influence of contextual factors and weekly periodisation within teams/players.

### Statistical analysis

Data were recorded for all match and training days and only the data of players who participated in that week’s match were included in the analysis of the team’s preparation for that game. To identify underlying associations between the training load variables, a Pearson product-moment correlation was calculated between sRPE/total distance, sRPE/HSR and total distance/HSR. Correlation coefficients were interpreted using the following classification (0.9 to 1.00: Very High, 0.70 to 0.90: High, 0.50 to 0.70: Moderate, 0.30 to 0.50: Low, .00 to 0.30: Negligible) ^[13]^. Average training load values for match day (MD) and each day prior (MD-1, MD-2, MD-3, MD-4, MD-5) were calculated for sRPE, total distance and HSR variables for all players included in the fixture for that week. To understand potential contextual factors that may have influenced the weekly prescription of training load in this sample, several other factors were included: opposition final season ranking (proxy measure for opposition standard), competition (Premiership, friendly, European, National Cup), result of previous fixture (win/loss/draw), surface type in next match (artificial turf or natural grass), and venue for the game (home/away). Linear mixed models were used to assess the differences between each preparation day on the respective training load metrics. Each of the contextual variables were included as fixed effects, with random effects included for each player within their respective teams. Analyses were undertaken using the lme4 package^[14]^ in RStudio (RStudio, Inc. Version 1.1.463). Estimated marginal means (EMMs) were calculated alongside corresponding 95% confidence intervals for each respective variable of interest, with differences assessed using effect sizes, confidence intervals and P-values using the emmeans package^[15]^. Effect sizes were interpreted using Cohen’s effect sizes (<0.2: Trivial, 0.2 – 0.5: Small, 0.5–0.8: Medium, >0.8: Large ^[16]^).

## Results

Over the study period, 128 332 days of training were recorded (excluding days off) across the six teams involved in the study. On examination of the training load variables, sRPE and total distance variables were deemed to have a moderate correlation (*r* =0.66 (95% CIs: 0.65–0.67)), sRPE and HSR a weak correlation (*r* =0.32 (95% CIs: 0.30–0.34)) and total distance and HSR a moderate correlation (*r* = 0.62 (95% CIs: 0.61–0.63)).

### Weekly distribution of training load

Across all training load metrics (sRPE, total distance and HSR), the match day represented the highest average per player with a mean of 513 AU (95% CIs: 398–629 AU), 4 209 m (95% CIs: 3 929–4 488 m) and 390 m (95% CIs: 146–633 m), respectively (Table 1). Match-day-1 (MD-1) represented the lowest training load of the five days preparation for games (101 AU (95% CIs: 0–216 AU), 1 047 m (95% CIs: 1 128–1 686 m) and 99 m (95% CIs: 0–343 m), respectively, while the day with the highest load outside of the match exposure was MD-4 (464 AU (95% CIs: 350–578 AU), 2 983 m (95% CIs: 2 704–3 262 m) and 234 m (95% CIs: 0–477 m) respectively shown in Table 1). Modelling the differences between match day exposure and each respective training day demonstrated large differences, with the exception of MD-4 which showed trivial differences between match day and training for sRPE and medium differences for total distance and HSR. The biggest differences were seen between match day and MD-1 across all variables (effect sizes: sRPE: 1.37, total distance: 1.42 and HSR 1.12 respectively, shown in Table 2).

### Standard of opposition

Using a team who finished 1^st^ or 2^nd^ during the season (high standard of opposition) as the reference group, only trivial effects on sRPE training load were seen in the days prior to a match (see Table S2). Similarly, for both the total distance and HSR, only trivial effects were found based on standard of opposition (see Tables S3/S4).

### Competition type

European fixtures were used as the reference category for comparisons between competition types. For sRPE, trivial differences were found between European, National Cup and Premiership fixtures. However, small negative effects were seen between European fixtures and friendlies (ES = −0.30, 95% CIs: −0.36 to −0.25, p<0.001) with players undertaking an average of 245 AU of training load per day on European weeks compared with 337 AU during the weeks of friendly games (see Tables S1/S2). For total distance, European fixtures demonstrated the lowest daily averages across all competition types (1 881 m), with small significant effects seen when compared to National Cup and Premiership fixtures (ES = −0.27 for both comparisons) but only trivial effects when compared with friendlies (see Tables S1/S3/S4). High-speed running demonstrated only trivial changes based on competition type. Follow-up analysis demonstrated that Premiership games had the highest match load for sRPE (Median: 560 AU, IQR: 300–720 AU), National Cup games demonstrated the highest median total distance covered in a game (Median: 4 519 m, IQR: 2 304–6 355 m) and Premiership games demonstrated the highest median HSR distances (Median: 240 m, IQR: 96–541 m: see Table S5).

### Previous result, surface type, and venue

The outcome of the previous game (win or loss) demonstrated only trivial effects on training load in the subsequent week across all training load measures (see Tables S2/S3/S4). The surface type on which the upcoming game would be played (natural grass or artificial turf) demonstrated only trivial effects on the training load in the preceding week across all training load measures (see Tables S2/S3/S4). Finally, the location of the upcoming game (home vs. away) demonstrated only trivial effects on training load across all three training load variables (see Tables S2/S3/S4).

## Discussion

This study is the first to objectively examine the distribution of training across a week in professional rugby union. The match day itself demonstrated the highest average load, while four days prior to the match (MD-4) demonstrated the greatest training stimulus, based on the sRPE, total distance, and HSR metrics. The day with the lowest training exposure and therefore the day with the largest effect size compared with match days was the day before a game (MD-1: effect sizes 1.12–1.42). When examining training prior to different competition types, European fixtures demonstrated lower average training loads for sRPE compared to friendlies and lower total distance values compared with Premiership and National Cup fixtures. Of the other remaining contextual factors considered, none demonstrated more than trivial changes in the average load per player in the days prior to a match.

### What is the structure of a training week?

It has been previously shown that professional rugby players train an average of 6 h 48 min (95% CIs: 6 h 30 min − 7 h 4 min) per week.^[7]^ While this is useful for understanding weekly volume, it is imperative to understand the intensity and how training is distributed throughout the week. The training structure of a professional rugby union team has previously been shown for the five days prior to competitive matchplay^[9]^; however, only weekly totals of training load for sRPE and GPS metrics were presented, meaning that the distribution of load within the individual sessions was not clear. The current study builds on the available research to demonstrate the typical distribution of training throughout the week. Given the highly attritional nature of professional rugby union, the frequency of matches is typically limited to one per week, usually with a minimum of a five-day turnaround between games. This study has shown that the greatest training stimulus in preparation for games is that of MD-4, which had an average sRPE of 464AU (350–578), average total distance of 2 983 m (2 704–3 262) and average HSR of 234 m (0–477) per athlete. Unsurprisingly, the day prior to a game (MD-1) had the lowest training stimulus. This reduced load on MD-1 aligns with evidence in rugby union, as well as football, reporting a decline in training load in the days prior to matches^[8, 9]^.

### Is the weekly structure between teams similar?

**Fig. 1:**
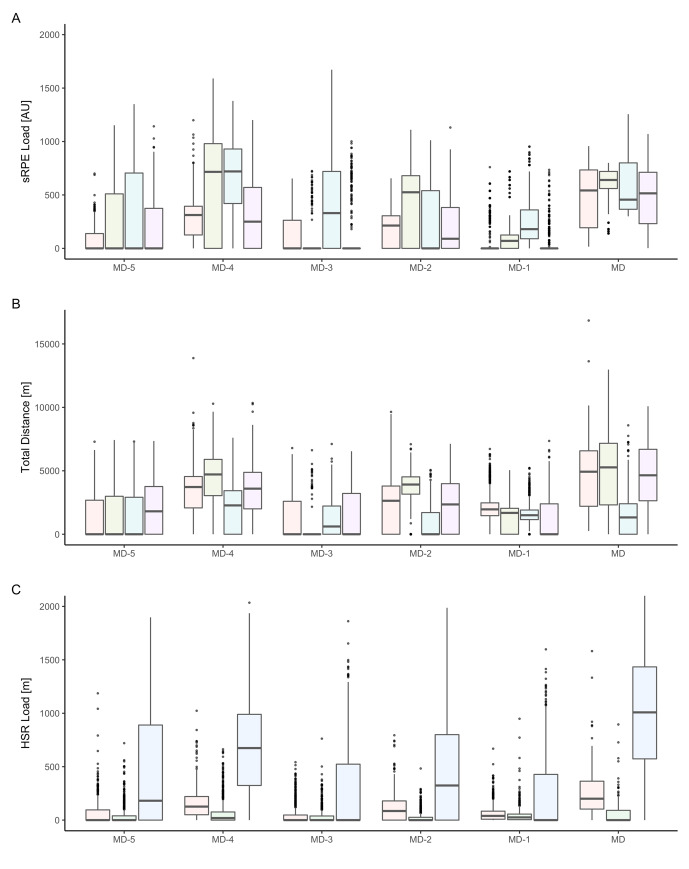
Club by club median player training load distribution across a week. A) session Rating of Perceived Exertion (sRPE) load B) total distance (TD) derived from Global Positioning System (GPS) C) High-speed running (HSR) derived from GPS. Each bar represents a different club. MD: Match Day, MD-1: 1 day prior to match, MD-2: 2days prior to match, MD-3: 3days prior to match, MD-4: 4days prior to match, MD-5: 5days prior to match. A) Only four clubs provided suitable data for sRPE and to avoid inflated or deflated scores, two clubs were removed from this analysis. B) one club provided unsuitable data and was removed from the TD analysis. C) two clubs provided unsuitable data and were removed from the HSR analysis. Medians include all players involved in matches in that week (irrespective of whether they partook in every session).

Fig. 1 demonstrates the differences between clubs in their approach to the weekly distribution of load and highlights the importance of including multiple teams in descriptive studies of this type. This is required to adequately portray the different conditioning strategies employed by staff and highlights the lack of a ‘one size fits all’ approach. Largely, the structure of the training week was similar between clubs, with some clubs employing a slightly different structure, electing to take rest days on different days. Previous studies have generally been limited to a single club, giving rise to the potential for club specific practices to influence the weekly structure.^[8, 9]^ This study included data from six teams across the same league and therefore decreases the likelihood of the results to be representative of just one club. One of the most substantial differences between clubs is that of the values associated with high-speed running. However, as outlined previously, this study used a HSR defined by each club, which included a range of values, for example, one club defining HSR as 40–70% Vmax, compared with others which included >70% Vmax.

### The influence of contextual factors on training load

Despite both empirical evidence in football^[10]^ and anecdotal evidence in rugby union in this study, contextual factors, including standard of opposition, previous result, surface type and venue, had only trivial effects on the training load of players. Of the included contextual factors, only the competition type demonstrated an effect, with European fixtures being preceded by the lowest average training loads per player per day for sRPE, total distance and HSR. As friendly fixtures are largely reserved for the pre-season period, the finding linked to sRPE is unsurprising, given that it has been previously shown that during the pre-season period, training volume is higher than in-season (9 h per week vs, 6 h 6 min per week).^[7]^ With the pre-season period designed to prepare players for the season, weekly training volumes will often be higher to improve fitness, giving rise to the higher sRPE load. Given the difference between the European fixtures and friendlies this was greater for sRPE and not total distance and HSR metrics, suggesting that either a greater amount of gym-based training is undertaken during the pre-season or the internal load produced by the external load is higher in pre-season due to lower fitness levels.^[7]^ For both of the GPS-based running metrics, significantly lower training was shown to be undertaken in the weeks prior to European fixtures compared with that of the Premiership and National Cup fixtures, which suggests a tapering strategy prior to important European fixtures. However, it is important to consider that in some cases, (when the outcome of a group in the competition is already decided), games become less important and therefore teams may use these weeks as an opportunity for lower training loads in anticipation of future fixtures.

### Which is the most physically demanding competition and why do external and internal loads not match up?

While not a primary aim of this study, the disparity between subjective internal load (sRPE) and objective external load is particularly interesting. The wide interquartile range suggests a broad spectrum of values are likely and the median values demonstrate that, based on sRPE and HSR, Premiership fixtures provide the highest match load stimulus. However, based on total distance, National Cup fixtures represent the highest match load stimulus. As the leading national and international competitions, it would be expected that the Premiership and European Cup would elicit the highest match loads and, therefore, the finding that the National Cup represented the highest total distance load was surprising. However, it is possible that the the weekly competition may influence squad selection and game-related factors that may be associated with increased ball in play time, greater time spent in offense vs. defence, and other patterns of play. Further work to understand why these differences occur would help to differentiate between match demands and their association with competition type. Compared with data from a similar cohort, the total distance values associated with match play (Median: 4 483 m, IQR:2 069–6 478) were lower than those previously reported (5 581 m ± 692 for forwards, 6 127 m ± 724 for backs).^[17]^ However, Roberts et al.^[17]^ normalised their data to 80 minutes of play per position, whereas in the context of this study, the data showed the average distance covered per player that partook in the game for any period of time, which explains the difference in the total distance covered.

### Limitations

A significant limitation of this study is the exclusion of collision-based metrics. Collision is a significant component of both training and match physical stimulus and therefore its omission is important to recognise. However, given the pilot work that was undertaken in advance of this study, the methods and consistency with which collision is collected across clubs was not considered sufficient to make broader conclusions than on a club-by-club basis.^[6]^ Secondly, the present study reported match data as the average value per player involved in the game for any period of time and therefore, includes players who had only played for short periods of time, as well as full matches. In addition, as this was a multi-club study where each club used the available technology in different ways, which led to differences in not only the definitions used in the HSR variables but also some clubs data being unusable due to certain metrics not being collected on certain days (e.g. sRPE on a match day). While this is a limitation of the study, given the multi-club nature and the desire to represent the data as used in daily practice, these methods were used and accounted for in any analysis through the use of generalised linear mixed models, including random effects for each player within their club.

## Conclusion

This study outlines the training distribution of teams in preparation for professional rugby union matches. Strengthened by the inclusion of data from multiple clubs, the generalisability of the data to other professional rugby environments is useful. As per other team sports, daily training load in the day preceding a match exposure is lowest across the week, with MD-4 demonstrating the highest training stimulus for running and perceived exertion, recognising that contact demand will likely differ substantially. There is a need for further investigation into the distribution of collision and contact training throughout the week, but this study provides an overview of weekly preparation for competitive professional rugby union.

## Supplementary Information



## Figures and Tables

**Table 1:** Mean (95% CIs) player training load distribution across a week

	MD-5	MD-4	MD-3	MD-2	MD-1	MD
**session Rating of Perceived Exertion (AU)**	185 (71–300)	464 (350–578)	160 (46–275)	241 (127–355)	101 (0–216)	513 (398–629)
**Total distance (metres)**	1 298 (1 019–1 577)	2 983 (2 704–3 262)	1 226 (947–1 505)	1 995 (1 716–2 274)	1 407 (1 128–1686)	4 209 (3 929–4 488)
**High-speed running (metres)**	142 (0–385)	234 (0–477)	108 (0–351)	155 (0–399)	59 (0–343)	390 (146–633)

MD, Match Day; MD-1, 1 day prior to match; MD-2; 2 days prior to match; MD-3, 3 days prior to match; MD-4, 4 days prior to match; MD-5, 5 days prior to match

**Table 2:** Effect size (95% CIs), P-value, and effect size interpretation of modelled difference between match day and each respective training day

	Measure	MD to MD-5	MD to MD-4	MD to MD-3	MD to MD-2	MD to MD-1
**session Rating of Perceived Exertion (AU)**	**Effect size**	1.09 (1.03–1.14)	0.16 (0.11–0.22)	1.17 (1.12–1.23)	0.90 (0.85–0.96)	1.37 (1.31–1.42)
	**P-value**	<0.001	<0.001	<0.001	<0.001	<0.001
	**Effect size interpretation**	Large	Small	Large	Large	Large

**Total distance (metres)**	**Effect size**	1.48 (1.44–1.52)	0.62 (0.58–0.66)	1.52 (1.48–1.56)	1.13 (1.09–1.17)	1.42 (1.38–1.46)
	**P-value**	<0.001	<0.001	<0.001	<0.001	<0.001
	**Effect size interpretation**	Large	Moderate	Large	Large	Large

**High-speed running (metres)**	**Effect size**	0.95 (0.91–1.00)	0.60 (0.55–0.64)	1.09 (1.04–1.13)	0.90 (0.85–0.94)	1.12 (1.07–1.16)
	**P-value**	<0.001	<0.001	<0.001	<0.001	<0.001
	**Effect size interpretation**	Large	Moderate	Large	Large	Large

MD, Match Day; MD-1, 1 day prior to match; MD-2; 2 days prior to match; MD-3, 3 days prior to match; MD-4, 4 days prior to match; MD-5, 5 days prior to match
