# Supplementary material for: Training and match load in professional rugby union: Do contextual factors influence the training week?
Source: S Afr J Sports Med. 2021 May 25;33(1):v33i1a9509. doi: 10.17159/2078-516X/2021/v33i1a9509 (PMC9924541; doi:10.17159/2078-516X/2021/v33i1a9509)
Supplement: Supplementary file 1 [file 2078-516X-33-v33i1a9509-s001.pdf]

Supplementary data:

Table S1: Mean daily training load per player.

	Measure	Session Rating of Perceived Exertion (AU)	Total Distance (metres)	High Speed Running (metres)
**Standard of Opposition**	1st / 2nd	305 (188-422)	2086 (1718- 2453)	192 (0-440)
	3rd / 4th	263 (146- 380)	2026 (1654-2398)	203 (0-452)
	5th / 6th	261 (143-379)	1984 (1606-2361)	171 (0-420)
	7th / 8th	295 (166-424)	1977 (1601-2352)	192 (0-440)
	9th / 10th	280 (163-398)	1919 (1542-2297)	176 (0-425)
	11th / 12th	289 (171-407)	2031 (1650-2413)	190 (0-438)
**Competition Type**	Europe	245 (136-355)	1881 (1607-2154)	174 (0-404)
	Friendly	337 (227-447)	2118 (1835-2400)	176 (0-407)
	National Cup	269 (161-378)	2403 (2139-2668)	207 (0-437)
	Premiership	259 (151-366)	2342 (2668-2596)	195 (0-425)
**Previous Result**	Loss	281 (184-378)	2217 (1922- 2387)	191 (0-391)
	Win	274 (177-371)	2155 (1985- 2449)	185 (0-398)
**Surface Type**	Artificial Turf	276 (179-373)	2312 (2077-2547)	188 (0-295)
	National Grass	279 (182-376)	2060 (1829-2291)	187 (0-294)
**Match Venue**	Away	287 (190- 384)	2250 (2017-2482)	191 (0-398)
	Home	269 (172-365)	2122 (1891-2354)	185 (0-392)

Table S2: Effect Size (95% CIs), P-Value, and effect size interpretation of modelled difference between reference group and comparison groups for session Rating of Perceived Exertion.

	Comparison Group	Effect Size (95% CIs)	P-Value	Effect Size Interpretation
**Standard of Opposition (Ref: 1st/2nd)**	3rd / 4th	0.14 (0.09-0.19)	**<0.001**	*Trivial*
	5th / 6th	0.15 (0.09-0.20)	**<0.001**	*Trivial*
	7th / 8th	0.08 (0.03-0.14)	**0.002**	*Trivial*
	9th / 10th	0.08 (0.03-0.14)	**0.003**	*Trivial*
	11th / 12th	0.05 (0.01-0.11)	0.076	*Trivial*
**Competition Type (Ref: Europe)**	Friendly	-0.30 (-0.36 to -0.25)	**<0.001**	***Small***
	National Cup	-0.08 (-0.14 to -0.02)	**0.014**	*Trivial*
	Premiership	-0.04 (-0.11 to 0.02)	0.182	*Trivial*
**Previous Result (Ref: Loss)**	Win	-0.02 (-0.05 to 0.01)	0.18	*Trivial*
**Surface Type (Ref: Artificial Turf)**	National Grass	-0.01 (-0.05 to 0.03)	0.61	*Trivial*
**Match Venue (Ref: Away)**	Home	0.06 (0.03-0.09)	**<0.001**	*Trivial*

Table S3: Effect Size (95% CIs), P-Value, and effect size interpretation of modelled difference between reference group and comparison groups for Total Distance.

	Comparison Group	Effect Size (95% CIs)	P-Value	Effect Size Interpretation
**Standard of Opposition (Ref: 1st/2nd)**	3rd / 4th	0.03 (-0.02 to 0.08)	0.23	*Trivial*
	5th / 6th	0.05 (-0.00 to 0.11)	**0.05**	*Trivial*
	7th / 8th	0.06 (0.00 to 0.11)	**0.03**	*Trivial*
	9th / 10th	0.09 (0.03 to 0.14)	**0.002**	*Trivial*
	11th / 12th	0.03 (-0.03 to 0.14)	0.34	*Trivial*
**Competition Type (Ref: Europe)**	Friendly	-0.12 (-0.16 to -0.08)	**<0.001**	*Trivial*
	National Cup	-0.27 (-0.32 to -0.21)	**<0.001**	***Small***
	Premiership	-0.27 (-0.29 to -0.18)	**<0.001**	***Small***
**Previous Result (Ref: Loss)**	Win	-0.03 (-0.06 to 0.00)	**0.023**	*Trivial*
**Surface Type (Ref: Artificial Turf)**	National Grass	0.13 (0.10 to 0.16)	**<0.001**	*Trivial*
**Match Venue (Ref: Away)**	Home	0.06 (0.04 to 0.09)	**<0.001**	*Trivial*

Table S4: Effect Size (95% CIs), P-Value, and effect size interpretation of modelled difference between reference group and comparison groups for High Speed Running.

	Comparison Group	Effect Size (95% CIs)	P-Value	Effect Size Interpretation
**Standard of Opposition (Ref: 1st/2nd)**	3rd / 4th	-0.04 (-0.09 to 0.01)	**0.08**	*Trivial*
	5th / 6th	0.08 (0.02 to 0.13)	**0.003**	*Trivial*
	7th / 8th	0.00 (-0.05 to 0.05)	0.97	*Trivial*
	9th / 10th	0.06 (0.01 to 0.11)	**0.03**	*Trivial*
	11th / 12th	0.01 (-0.04 to 0.06)	0.97	*Trivial*
**Competition Type (Ref: Europe)**	Friendly	-0.01 (-0.06 to 0.04)	0.715	*Trivial*
	National Cup	-0.13 (-0.18 to -0.07)	**<0.001**	*Trivial*
	Premiership	-0.08 (-0.14 to -0.03)	**0.004**	*Trivial*
**Previous Result (Ref: Loss)**	Win	-0.03 (-0.06 to 0.01)	0.102	*Trivial*
**Surface Type (Ref: Artificial Turf)**	National Grass	0.00 (-0.03 to 0.04)	0.83	*Trivial*
**Match Venue (Ref: Away)**	Home	0.02 (-0.01 to 0.05)	0.12	*Trivial*

Table S5: Median (Interquartile range) player match day load values for each respective competition types.

	Measure	Session Rating of Perceived Exertion (AU)	Total Distance (metres)	High Speed Running (metres)
**Competition Type**	Europe	456 (192-640)	4407 (2180-6214)	221 (92-439)
	Friendly	480 (320-720)	3574 (2546-4862)	188 (76-453)
	National Cup	490 (336-704)	4519 (2304-6355)	200 (90-470)
	Premiership	560 (300-720)	4483 (2069-6478)	240 (96-541)
